# Impact of Ghrelin‐Depleting Gastrectomy on Long‐Term Endocrine and Metabolic Health With a Focus on Skeletal Muscle and Bone Mineral Content

**DOI:** 10.1002/ags3.70055

**Published:** 2025-06-23

**Authors:** Hiroki Harada, Takuya Goto, Keishi Yamashita, Hiroyuki Minoura, Kota Okuno, Shohei Fujita, Mikiko Sakuraya, Tadashi Higuchi, Koshi Kumagai, Naoki Hiki

**Affiliations:** ^1^ Department of Upper Gastrointestinal Surgery Kitasato University School of Medicine Sagamihara Kanagawa Japan; ^2^ Department of Gastrointestinal Surgery Yamato Municipal Hospital Yamato Kanagawa Japan; ^3^ Division of Advanced Surgical Oncology, Department of Research and Development Center for New Medical Frontiers Kitasato University School of Medicine Sagamihara Kanagawa Japan

**Keywords:** bone mineral content, gastrectomy, gastric cancer, ghrelin, IGF‐1, skeletal muscle mass

## Abstract

**Background:**

Advances in diagnostic and surgical techniques have improved survival rates for gastric cancer patients. However, gastrectomy involving ghrelin‐secreting regions of the upper gastric greater curvature can lead to long‐term endocrine and metabolic disturbances, including reductions in serum ghrelin and insulin‐like growth factor‐1 (IGF‐1), potentially contributing to skeletal muscle and bone mineral loss.

**Methods:**

This prospective observational study included 35 gastric cancer patients who underwent gastrectomy between 2016 and 2018, with follow‐up for 3–5 years. Patients were categorized into ghrelin‐depleted (total or proximal gastrectomy) and ghrelin‐preserved (distal gastrectomy) groups. Serum desacyl‐ghrelin, IGF‐1, and insulin‐like growth factor‐binding protein‐3 (IGFBP‐3) levels were measured, and skeletal muscle mass and bone mineral content were assessed.

**Results:**

The ghrelin‐depleted group exhibited significantly lower serum desacyl‐ghrelin (56.9 ± 27.9 vs. 111.2 ± 54.8 fmol/mL, *p* = 0.0006), skeletal muscle mass (87.7% ± 2.1% vs. 95.1% ± 2.4%, *p* = 0.0229), and bone mineral content (90.9% ± 13.0% vs. 99.5% ± 6.3%, *p* = 0.0249). Additionally, IGF‐1 levels showed a significant positive correlation with skeletal muscle mass (*r* = 0.53, *p* = 0.020). While the correlation between IGF‐1 and bone mineral content did not reach statistical significance, a positive trend was observed (*r* = 0.44, *p* = 0.062).

**Conclusion:**

Gastrectomy involving resection of ghrelin‐rich regions leads to long‐term reductions in serum desacyl‐ghrelin levels, adversely affecting skeletal muscle mass and bone mineral content. These findings highlight the importance of considering the endocrine consequences when selecting surgical procedures.

## Graphical abstract

This study investigates the long‐term endocrine and metabolic consequences of ghrelin‐depleted gastrectomy, with a focus on skeletal muscle mass and bone mineral content. Sustained reductions in serum desacyl‐ghrelin were linked to lower IGF‐1 levels, resulting in significant loss of muscle and bone. These findings underscore the importance of preserving ghrelin‐rich regions during gastric cancer surgery to maintain long‐term metabolic health.

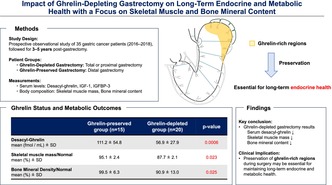

## 1 Introduction

Advances in diagnostic and surgical techniques for gastric cancer have improved the survival rates of patients. In Japan, the 5‐year overall survival rate for patients with resectable gastric cancer is 71.3%, exceeding 90% for those with Stage I disease. Consequently, the number of long‐term survivors following gastrectomy for gastric cancer has been increasing [1, 2]. Consequently, post‐gastrectomy sequelae have attracted significant attention.

Patients undergoing gastrectomy experience structural changes, such as reduced food intake due to a diminished gastric reservoir capacity, along with functional changes, including impaired digestion and lipid absorption. These changes are considered the primary causes of postoperative weight loss, skeletal muscle loss, and decreased bone mineral content [3, 4, 5]. However, there are cases where patients exhibit long‐term reductions in body weight, skeletal muscle mass, and bone mineral density despite improvements in dietary intake.

Ghrelin, a peptide hormone predominantly secreted by the gastric fundus, plays a pivotal role in appetite regulation, energy homeostasis, and the stimulation of the growth hormone (GH)/insulin‐like growth factor‐1 (IGF‐1) axis [6]. Its secretion is notably abundant in the fundus and upper gastric body, whereas the duodenum and other parts of the gastrointestinal tract contribute less significantly to overall ghrelin levels [7]. Post‐gastrectomy patients often experience a significant reduction in serum ghrelin levels, particularly when surgeries involve regions with high ghrelin‐secreting cell density, such as total gastrectomy (TG) [8, 9].

Ghrelin's downstream effects on the GH/IGF‐1 axis are critical for maintaining skeletal muscle and regulating bone metabolism [10]. IGF‐1, primarily synthesized in the liver under GH stimulation, exerts anabolic effects on skeletal muscle and contributes to bone mineralization. Its bioavailability is modulated by insulin‐like growth factor‐binding protein‐3 (IGFBP3), which stabilizes and transports IGF‐1 in the circulation [11]. Consequently, disruptions in the ghrelin‐GH/IGF‐1‐IGFBP3 axis due to gastrectomy may significantly impact long‐term skeletal muscle mass and bone mineral content. However, long‐term postoperative studies focusing on the relationship between serum desacyl‐ghrelin levels, GH/IGF‐1 axis activity, IGFBP3 levels, and changes in skeletal muscle mass and bone mineral content remain scarce.

This study aims to investigate whether gastrectomy in ghrelin‐rich regions results in a sustained decrease in serum desacyl‐ghrelin levels compared with resection in ghrelin‐poor regions. Additionally, it explores the downstream effects on the GH/IGF‐1 axis, IGFBP3 levels, skeletal muscle mass, and bone mineral content in long‐term postoperative patients.

## 2 Patients and Methods

### 2.1 Patients

This prospective observational study included patients diagnosed with gastric cancer who underwent gastrectomy at Kitasato University Hospital between January 2016 and August 2018. Eligible patients were those who completed long‐term follow‐up for 3 to 5 years post‐surgery. This follow‐up period was selected to minimize the influence of early postoperative fluctuations in dietary intake and nutritional status, ensuring evaluation under stable conditions. Furthermore, the study aimed to assess the long‐term and sustained effects on endocrine markers, including ghrelin, IGF‐1, and IGFBP‐3, rather than transient postoperative changes. Written informed consent was obtained from all participants in accordance with institutional review board requirements (Approval number: B20‐211).

### 2.2 Surgical Procedures

Surgical procedures included TG, proximal gastrectomy (PG), and distal gastrectomy (DG). Patients were categorized based on the extent and region of gastric resection into two groups: ghrelin‐rich resection group (ghrelin‐depleted group), consisting of those who underwent TG or PG, and ghrelin‐poor resection group (ghrelin‐preserved group), which included patients who underwent DG.

### 2.3 Serum Desacyl‐Ghrelin

Serum desacyl‐ghrelin levels were measured using the Desacyl‐Ghrelin ELISA Kit (SCETI Co. Ltd., Tokyo, Japan) following the manufacturer's protocol. Blood samples were collected in the morning after an overnight fast during routine postoperative follow‐up visits. Serum was separated by centrifugation at 3000 rpm for 15 min at 4°C and stored at −80°C until analysis. The assay had a detection range of 1.0–100.0 pg/mL, and all samples were analyzed in duplicate to ensure accuracy.

### 2.4 Serum IGF‐1

Serum IGF‐1 levels were measured using an electrochemiluminescence immunoassay (ECLIA, JaICA). The assay was performed using a fully automated analyzer following the manufacturer's instructions. Blood samples were collected in the fasting state, processed, and stored under identical conditions. IGF‐1 values were normalized based on age‐ and sex‐specific reference ranges provided by the manufacturer, ensuring comparability across the study population.

### 2.5 Serum IGFBP‐3

Serum IGFBP‐3 levels were determined using the IGFBP‐3 ELISA Kit (R&D Systems, USA). The assay was conducted according to the manufacturer's guidelines. Samples were thawed at room temperature, diluted appropriately, and analyzed in duplicate for consistency. The ELISA had a sensitivity of 0.1 ng/mL and a range of 0.1–10.0 μg/mL. Internal quality controls were included in each batch to verify assay performance.

### 2.6 Body Composition

Body composition, including skeletal muscle mass and bone mineral content, was evaluated using the InBody system. The results from the InBody analysis were compared with normative age‐ and sex‐matched reference data to assess deviations and postoperative impacts. These measurements were conducted under standardized conditions to minimize variability. Importantly, serum sampling (for desacyl‐ghrelin and IGF‐1) and body composition assessments were performed on the same day during a scheduled postoperative follow‐up visit, ensuring consistency in the timing of measurements.

### 2.7 Patient Background and Clinical Variables

Baseline patient characteristics, including age, sex, comorbidities, and body mass index (BMI), were collected. Tumor‐related pathological factors, such as location, and clinical‐pathological stage, were also recorded. Additionally, surgical factors, including procedure type, operative time, blood loss, and postoperative complications, were evaluated. The severity of postoperative complications was classified according to the Clavien‐Dindo classification system [12].

### 2.8 Statistical Analysis

Differences between groups were assessed using student's *t*‐test or the Mann–Whitney *U* test, depending on the normality of data distribution. Correlations between serum endocrine parameters (desacyl‐ghrelin, IGF‐1, IGFBP3) and skeletal muscle mass or bone mineral content were analyzed using Pearson's correlation coefficient. Statistical significance was set at *p* < 0.05 for all analyses. To account for potential confounding factors, we also performed multivariate linear regression analyses as dependent variables. In each model, surgical procedure (ghrelin‐depleted vs. preserved), pathological stage (Stage II/III vs. I), and postoperative adjuvant chemotherapy were included as predictors. Statistical analysis was conducted using SAS software (JMP Pro 16, SAS Institute, Cary, NC, USA).

## 3 Results

### 3.1 Patient Characteristics

The study included 35 patients with a follow‐up period of 3–5 years post‐surgery. Baseline characteristics, including age, sex distribution, BMI, and body weight, were comparable between the ghrelin‐preserved and ghrelin‐depleted groups, with no significant differences observed. In contrast, the incidence of postoperative complications was significantly higher in the ghrelin‐depleted group (35%, 7/20) compared with the ghrelin‐preserved group (15%, 3/20) (*p* = 0.045). Significant differences were also noted in pathological stage (*p* = 0.032) and the rate of postoperative adjuvant therapy (*p* = 0.021) (Table 1).

**TABLE 1:** Patient characteristics.

Variables	Ghrelin‐preserved group (*n* = 15)	Ghrelin‐depleted group (*n* = 20)	*p*‐value
Age at surgery, years, median (range)	71 (55–85)	69.5 (52–81)	0.9734
Sex			
Male/Female	6/9	14/6	0.0759
Body weight, kg, median (range)	54.8 (47.8–72.1)	63 (36–74)	0.3865
Body mass index, kg/m2, median (range)	22.8 (18.5–36.0)	22.3 (14.6–27.8)	0.6304
Serum albumin level, g/dl, median (range)	4.4 (3.1–4.8)	4.15 (2.5–5)	0.2482
Atrophic gastritis			
Absence/Presence	4/11	4/16	0.6421
Anastomotic procedures			
Billroth‐I/Roux‐en‐Y	9/6	—	
Surgical procedure			
Total gastrectomy/Proximal gastrectomy	—	16/4	
Postoperative complication (CDc grade > 1)			
Absence/Presence	14/1	8/12	0.0012
Pathological stage (pStage)			
pStage I/II/III	14/1/0	10/6/4	0.0215
Postoperative adjuvant chemotherapy			
Absence/Presence	14/1	11/9	0.0130

Abbreviation: CDC, Clavien‐Dindo classification.

### 3.2 Nutritional Indicators and Symptoms

In the ghrelin‐depleted group, body weight loss was greater (median: −10.35 kg vs. −6.1 kg), with a trend toward significance, though the difference was not statistically significant (*p* = 0.0675). No significant differences were observed between the two groups in terms of body weight or serum albumin levels. The postoperative follow‐up period was a median of 51 months (range: 36–60 months) in the ghrelin‐preserved group and 52 months (range: 35–63 months) in the ghrelin‐depleted group, with no significant difference between the groups (*p* = 0.8474) (Table 2).

**TABLE 2:** Nutritional indicators and symptoms at the time of specimen collection.

Variables	Ghrelin‐preserved group (*n* = 15)	Ghrelin‐depleted group (*n* = 20)	*p*‐value
Body weight, kg, median (range)	52.5 (39.3–64.4)	50.35 (34.5–63.5)	0.6304
Body weight loss, kg, median	−6.1	−10.35	0.0675
Serum albumin level, g/dl, median (range)	4.3 (3.8–4.9)	4.15 (3.6–4.7)	0.4310
Serum albumin loss, g/dl, median (range)	0	−0.1	0.2257
Post gastrectomy gastrointestinal symptoms, *n* (%)	4 (26.7)	8 (40)	0.4109
Dumping symptoms, *n* (%)	5 (33.3)	4 (20)	0.3718
Reflux symptoms, *n* (%)	1 (6.7)	7 (35)	0.0482
Diarrhea symptoms, *n* (%)	4 (26.7)	8 (40)	0.4109
Time point of postoperative measurement, months, median (range)	51 (36–60)	52 (35–63)	0.8474

### 3.3 Serum Desacyl‐Ghrelin Levels

Serum desacyl‐ghrelin levels were significantly lower in the ghrelin‐depleted group (56.9 ± 27.9 fmol/mL) than the ghrelin‐preserved group (111.2 ± 54.8 fmol/mL) (*p* = 0.0006). This reduction remained consistent throughout the follow‐up period, suggesting long‐term suppression of desacyl‐ghrelin secretion in the ghrelin‐depleted group (Table 3).

**TABLE 3:** Hormone levels at the time of specimen collection.

Variables	Ghrelin‐preserved group (*n* = 15)	Ghrelin‐depleted group (*n* = 20)	*p*‐value
Desacyl‐ghrelin, mean (fmol/mL) ± SD	111.2 ± 54.8	56.9 ± 27.9	0.0006
IGF‐1, mean (nmol/L) ± SD	81.1 ± 20.5	79.4 ± 20.2	0.8064
IGF‐1 levels as a percentage of normal values, mean (%) ± SD	65.7 ± 14.7	61.4 ± 13.1	0.3700
IGFBP3, mean (ng/mL) ± SD	1631.8 ± 519.7	1539.6 ± 501.5	0.5996
IGF‐1 / IGFBP3, mean ± SD	0.057 ± 0.028	0.060 ± 0.030	0.7833

Abbreviations: IGF‐1, Insulin‐like growth factor‐1; IGFBP3, Insulin‐like growth factor‐binding protein‐3; SD, Standard deviation.

**FIGURE 1:**
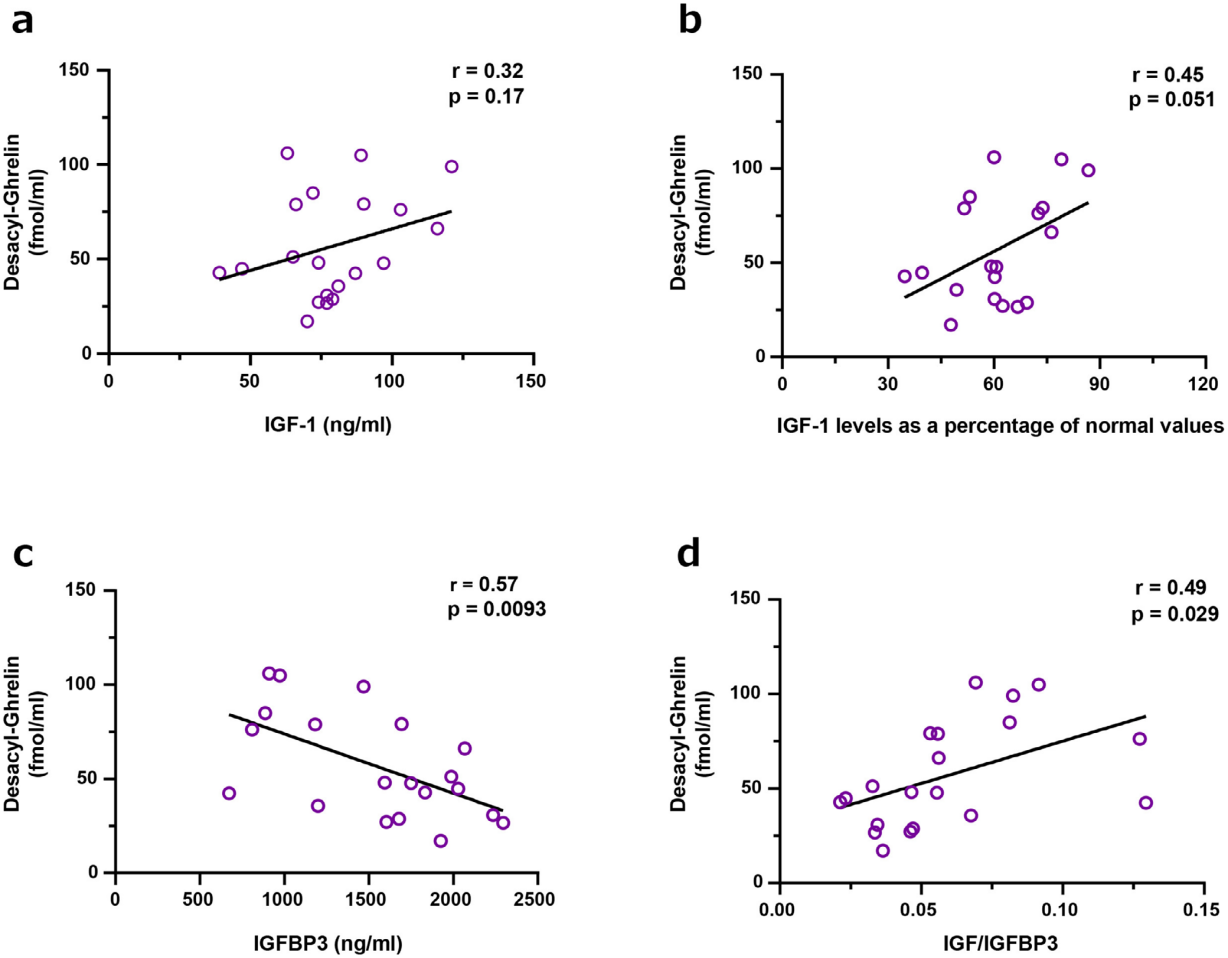
Desacyl‐ghrelin levels and correlations with IGF‐1 axis components. (a) Serum desacyl‐ghrelin concentrations measured 3–5 years after surgery, compared between the ghrelin‐preserved and ghrelin‐depleted groups. (b) Scatter plot illustrating the relationship between desacyl‐ghrelin and IGF‐1 concentrations. (c) Scatter plot illustrating the relationship between desacyl‐ghrelin and IGFBP‐3 concentrations. (d) Scatter plot illustrating the relationship between desacyl‐ghrelin and the IGF‐1/IGFBP‐3 ratio, a surrogate of IGF‐1 bioavailability.

Correlation analysis between desacyl‐ghrelin levels and hormonal parameters revealed a positive correlation trend with IGF‐1, although the result was not statistically significant (*r* = 0.32, *p* = 0.17) (Figure 1a,b). In contrast, a significant negative correlation was observed with IGFBP3 (*r* = −0.57, *p* = 0.0093), and a significant positive correlation was found with the IGF‐1/IGFBP3 ratio (*r* = 0.49, *p* = 0.029) (Figure 1c,d).

### 3.4 Skeletal Muscle Mass

**FIGURE 2:**
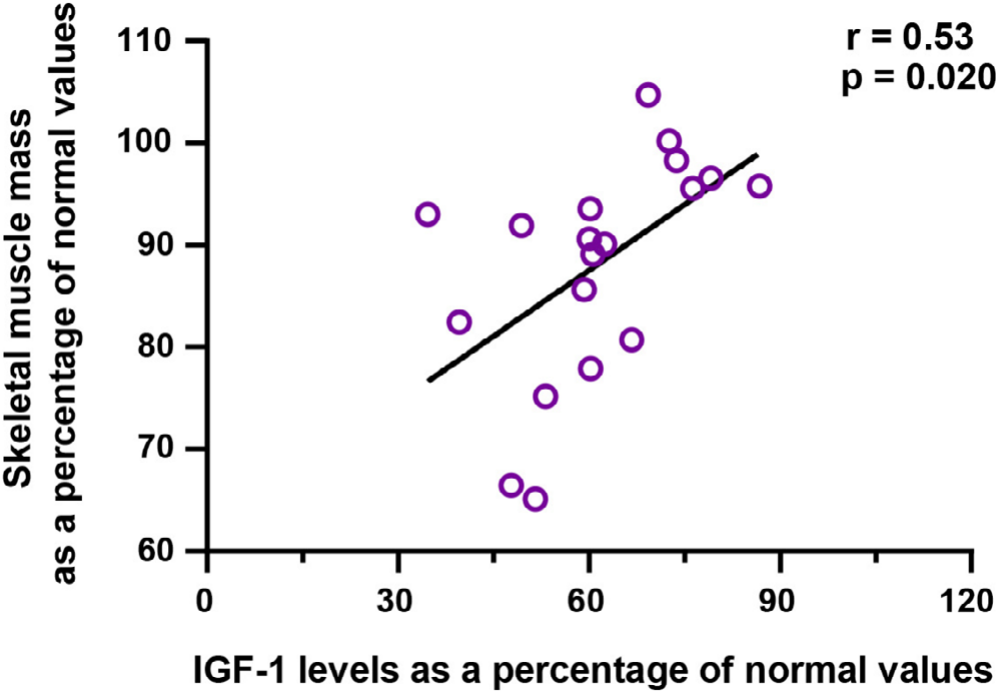
Impact of IGF‐1 on Skeletal Muscle Mass. IGF‐1 levels and skeletal muscle mass expressed as a percentage of normal values. The figure illustrates the correlation between circulating IGF‐1 levels and skeletal muscle maintenance, highlighting the impact of IGF‐1 on muscle mass preservation across different experimental conditions.

Skeletal muscle mass was significantly lower in the ghrelin‐depleted group compared with the ghrelin‐preserved group (percentage of normal values: 87.7% ± 2.1% vs. 95.1% ± 2.4%, *p* = 0.0229) (Table 4). Furthermore, skeletal muscle mass showed a significant positive correlation with IGF‐1 levels (*r* = 0.53, *p* = 0.020) (Figure 2). These findings suggest that IGF‐1 plays an important role in maintaining muscle mass.

**TABLE 4:** Body composition values at the time of specimen collection.

Variables	Ghrelin‐preserved group (*n* = 15)	Ghrelin‐depleted group (*n* = 20)	*p*‐value
Skeletal muscle mass, mean (kg) ± SD	22.8 ± 4.8	23.4 ± 4.4	0.7255
Skeletal muscle mass as a percentage of normal values, mean (%) ± SD	95.1 ± 2.4	87.7 ± 2.1	0.0229
Bone mineral content, mean (kg) ± SD	2.42 ± 0.09	2.43 ± 0.08	0.8959
Bone mineral content as a percentage of normal values, mean (%) ± SD	99.5 ± 6.3	90.9 ± 13.0	0.0249

Abbreviation: SD, Standard deviation.

### 3.5 Bone Mineral Content

**FIGURE 3:**
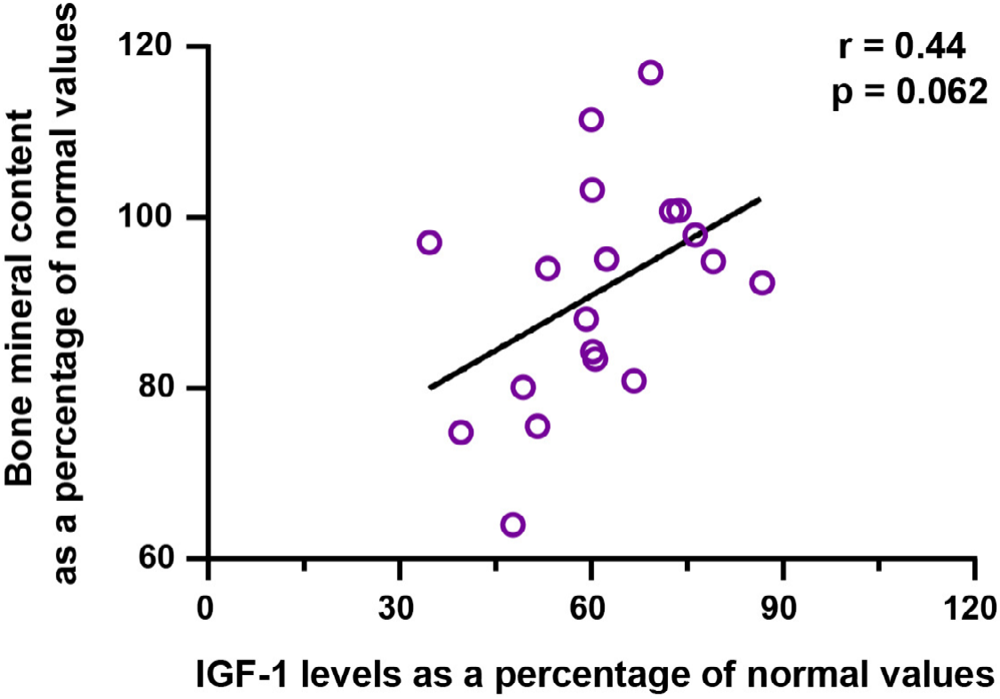
Bone Mineral Content and IGF‐1 Pathway. IGF‐1 levels and bone mineral content expressed as a percentage of normal values. The figure demonstrates the correlation between IGF‐1 and bone metabolism, suggesting a potential role of IGF‐1 in maintaining skeletal integrity.

Bone mineral content (as a percentage of normal values) was significantly lower in the ghrelin‐depleted group (90.9% ± 13.0%) compared with the ghrelin‐preserved group (99.5% ± 6.3%, *p* = 0.0249) (Table 4). Furthermore, bone mineral content showed a positive correlation trend with IGF‐1 levels, although it did not reach statistical significance (*r* = 0.44, *p* = 0.062) (Figure 3).

### 3.6 Multivariate Analysis Adjusting for Stage and Chemotherapy

Multivariate linear regression analysis showed that ghrelin‐depleting surgery was independently associated with both lower skeletal muscle mass (Estimate = −3.95, *p* = 0.0416) and reduced serum desacyl‐ghrelin concentration (Estimate = −28.27, *p* = 0.0018) (Table 5), even after adjusting for pathological stage and adjuvant chemotherapy. Neither pathological stage nor chemotherapy had a significant effect in either model.

**TABLE 5:** Multivariate Linear Regression Analysis.

	Skeletal muscle mass (% of ideal body weight)				Serum desacyl‐ghrelin concentration (fmol/mL)			
Variable (predictor)	Estimate (β)	Standard error	*t*‐value	*p*‐value	Estimate (β)	Standard error	*t*‐value	*p*‐value
Intercept	91.89	2.65	34.70	< 0.0001	91.46	11.76	7.78	< 0.0001
Surgical procedure (Ghrelin‐preserved group vs. Ghrelin‐depleted group)	+3.95	1.86	2.13	0.0416	+28.27	8.24	3.43	0.0018
Pathological stage II vs. I	−1.72	7.03	−0.24	0.8085	−35.13	31.22	−1.13	0.2694
Pathological stage III vs. I	+1.71	3.89	0.44	0.6635	+16.10	17.27	0.93	0.3587
Postoperative adjuvant chemotherapy	+0.94	5.18	0.18	0.8570	+26.71	23.01	1.16	0.2549

## 4 Discussion

This study provides novel insights into the long‐term metabolic consequences of gastrectomy, emphasizing the effects on serum desacyl‐ghrelin, GH/IGF‐1 axis, skeletal muscle mass, and bone mineral content. Three major findings emerged from our analysis. First, gastrectomy in ghrelin‐rich regions results in persistent suppression of serum desacyl‐ghrelin levels, with significant downstream effects on endocrine signaling. Second, IGF‐1 and IGFBP‐3 levels were closely associated with ghrelin activity, and skeletal muscle mass was significantly reduced in the ghrelin‐depleted group, showing a strong correlation. Third, bone mineral content also tended to correlate with decreased IGF‐1 levels and was significantly reduced in the ghrelin‐depleted group. These findings collectively highlight the multifaceted endocrine and metabolic consequences of surgical intervention in ghrelin‐rich regions.

The first major finding underscores the critical role of ghrelin in maintaining endocrine balance post‐gastrectomy. Ghrelin acts directly on the pituitary gland to stimulate GH release, which in turn drives IGF‐1 synthesis in the liver [6]. The persistent and significant reduction in serum desacyl‐ghrelin observed in patients who underwent gastrectomy in ghrelin‐rich regions suggests a lasting impairment in this endocrine pathway. Previous studies have shown that ghrelin levels are closely linked to appetite regulation, energy expenditure, and overall metabolic health [6, 7]. Our results reinforce the need for tailored nutritional and hormonal interventions in patients undergoing extensive gastric resections to mitigate these effects. Although the ghrelin‐depleted group had a higher proportion of advanced pathological stage and received more adjuvant chemotherapy (Table 1), multivariate regression analysis demonstrated that surgical procedure was independently associated with both reduced serum desacyl‐ghrelin levels and decreased skeletal muscle mass. Pathological stage and chemotherapy did not show significant associations in either model. These findings suggest that ghrelin depletion itself plays a key role in long‐term metabolic decline.

It is possible that postoperative ghrelin levels gradually recover over time, particularly after distal gastrectomy. However, previous studies have shown that such recovery is limited or absent after total gastrectomy, even years after surgery [13]. In our cohort, the timing of blood collection was comparable between surgical groups (Table 2), and the TG/PG group still showed significantly lower desacyl‐ghrelin levels at 3–5 years postoperatively. Therefore, we believe that delayed recovery of ghrelin did not substantially confound our findings.

Importantly, these hormonal changes had meaningful clinical consequences. The second key observation relates to the relationship between IGF‐1 levels and skeletal muscle mass. IGF‐1 is well‐known for its anabolic effects on skeletal muscle, primarily through the activation of the PI3K/Akt signaling pathway [14]. Reduced IGF‐1 levels in the ghrelin‐depleted group were strongly correlated with diminished skeletal muscle mass, highlighting the endocrine pathway's role in preserving muscle protein synthesis and preventing muscle catabolism. This finding aligns with earlier studies reporting significant muscle mass loss in patients following TG compared with DG. Clinicians must consider postoperative supplementation strategies, including IGF‐1 analogs, to prevent muscle wasting and enhance functional recovery [15].

Furthermore, IGF‐1 plays a central role in the growth and regeneration of skeletal muscle, primarily through signaling mediated by the IGF‐1 receptor. This signaling pathway promotes muscle cell proliferation and differentiation, thereby increasing muscle protein synthesis. Additionally, IGF‐1 prevents muscle atrophy and contributes to the maintenance of muscle homeostasis [16]. Although GH is the primary upstream regulator of IGF‐1, its secretion is influenced by numerous factors such as sleep, stress, and fasting, and it exhibits pulsatile and diurnal variation. For this reason, standardized sampling protocols are essential for reliable GH measurement. In this retrospective study, such protocols were not feasible, and the available serum samples were not collected under controlled conditions. Therefore, we used IGF‐1 as a stable surrogate marker for GH activity, as commonly recommended in clinical and research settings [17, 18]. In patients following TG, the decline in IGF‐1 levels is thought to result from impaired nutrient absorption and alterations in hormone secretion. This leads to a reduction in muscle protein synthesis and an acceleration of muscle breakdown, potentially resulting in muscle mass loss. Therefore, maintaining and enhancing the IGF‐1 pathway could represent a promising strategy to prevent muscle mass loss in patients after TG.

The third significant finding highlights the impact of IGF‐1 reductions on bone mineral content. IGF‐1 is a key regulator of bone metabolism, influencing both osteoblast proliferation and collagen synthesis [19]. IGF‐1 plays a central role in bone growth and remodeling. Specifically, it promotes the proliferation of osteoblasts and increases the synthesis of collagen, a major component of the bone matrix [20]. Furthermore, IGF‐1 inhibits bone resorption and contributes to the maintenance of bone mineral density. These actions are essential for preserving bone strength and quality [21].

In the present study, a significant reduction in bone mineral density was observed in the ghrelin‐depleted group (Table 4), which is believed to be closely associated with reduced IGF‐1 levels (Figure 3). Previous studies have also reported that decreased IGF‐1 levels are linked to reduced bone mineral density and an increased risk of fractures [10]. This observation suggests that disruption of the GH/IGF‐1 axis not only affects muscle mass but also compromises bone health over time. Monitoring bone density and implementing proactive measures such as calcium and vitamin D supplementation are essential components of long‐term care. However, to address the root cause of these metabolic consequences, surgical strategies must be reconsidered.

Emerging evidence supports the idea that preserving ghrelin‐secreting regions during gastrectomy may help maintain endocrine and metabolic health. Because serial ghrelin measurements were not uniformly available, we used surgical procedure as a proxy for ghrelin depletion, consistent with previous reports [22, 23]. To reinforce this approach, we also conducted correlation analyses using actual postoperative desacyl‐ghrelin levels, which supported its physiological relevance (Figures 1, 2, 3). Existing studies suggest the importance of preserving the proximal stomach, where ghrelin‐secreting cells are most abundant. For instance, Pokrowiecka et al. reported that preserving the gastric fundus is critical for maintaining postoperative ghrelin levels [22]. Additionally, another study demonstrated that changes in ghrelin levels after gastrectomy are dependent on the type and extent of surgery [23]. These insights highlight the need for developing and adopting surgical techniques that minimize disruption to ghrelin‐secreting regions, particularly in cases where the extent of gastrectomy can be modified without compromising oncological outcomes.

In line with this rationale, we are currently developing a novel surgical procedure that preserves the ghrelin‐secreting region. This procedure, termed appetite‐preserving gastrectomy, has been reported to maintain serum ghrelin levels, skeletal muscle mass, and appetite in patients with esophagogastric junction cancer [24]. Moving forward, we plan to increase the number of cases and investigate bone mineral density and other biomarkers.

Despite these important findings, our study has several limitations. First, the relatively small sample size may limit the generalizability of the results. A larger cohort with extended follow‐up is necessary to validate these findings further. Second, preoperative endocrine and body composition data were unavailable, preventing direct comparisons between pre‐ and postoperative states. Third, while this study highlights significant correlations between endocrine markers and skeletal muscle/bone parameters, causal relationships remain speculative. In addition, although desacyl‐ghrelin levels were significantly lower in the ghrelin‐depleted group, no significant group differences were observed in IGF‐1, IGFBP‐3, or the IGF‐1/IGFBP‐3 ratio. This discrepancy may reflect physiological adaptation in the chronic postoperative phase or the influence of other factors such as nutrition, insulin sensitivity, and liver function on IGF‐1 regulation. It is also possible that early postoperative differences had normalized by the time of assessment at 3–5 years.

In conclusion, gastrectomy in ghrelin‐rich regions leads to persistent and significant reductions in serum desacyl‐ghrelin levels, adversely affecting skeletal muscle mass and bone mineral content. These findings underscore the importance of preserving endocrine function during gastric resections whenever possible and highlight the need for postoperative interventions to mitigate endocrine and metabolic disruptions. Future research should focus on larger, multicenter studies to validate these findings and explore targeted therapeutic strategies aimed at optimizing long‐term outcomes in post‐gastrectomy patients.

## Author Contributions

**Hiroki Harada:** conceptualization, methodology, formal analysis, writing – original draft, writing – review and editing. **Takuya Goto:** conceptualization, data curation, formal analysis, methodology. **Keishi Yamashita:** conceptualization, supervision. **Hiroyuki Minoura:** data curation, investigation. **Kota Okuno:** data curation, investigation. **Shohei Fujita:** data curation, writing – review and editing. **Mikiko Sakuraya:** writing – review and editing, resources. **Tadashi Higuchi:** resources, writing – review and editing, visualization. **Koshi Kumagai:** supervision, writing – review and editing. **Naoki Hiki:** conceptualization, supervision, writing – review and editing, writing – original draft.

## Ethics Statement

All procedures were conducted in accordance with the ethical standards of the responsible institutional and national committees on human experimentation and with the 1964 Helsinki Declaration and its later amendments. This study was approved by the Institutional Review Board of Kitasato University (Approval No. B20‐211).

## Consent

Informed consent, or a substitute for it, was obtained from all patients for inclusion in this study.

## Conflicts of Interest

Drs. Hiroki Harada, Keishi Yamashita, and Naoki Hiki serve as Editorial Board members for the Annals of Gastroenterological Surgery. Koshi Kumagai has financial relationships involving honoraria from Abbott Japan LLC, The Japan Surgical Society, Medtronic Japan Co. Ltd., Miyarisan Pharmaceutical Co. Ltd., Nobelpharma Co. Ltd., Nutri Co. Ltd., Zeon Medical Inc., and Zeria Pharmaceutical Co. Ltd. outside the submitted work. The authors declare that there are no conflicts of interest directly related to the content of this article.
